# Screening for Small Molecule Inhibitors of BMP-Induced Osteoblastic Differentiation from Indonesian Marine Invertebrates

**DOI:** 10.3390/md18120606

**Published:** 2020-11-30

**Authors:** Hiroyuki Yamazaki, Satoshi Ohte, Henki Rotinsulu, Defny S. Wewengkang, Deiske A. Sumilat, Delfly B. Abdjul, Wilmar Maarisit, Magie M. Kapojos, Michio Namikoshi, Takenobu Katagiri, Hiroshi Tomoda, Ryuji Uchida

**Affiliations:** 1 Faculty of Pharmaceutical Sciences, Tohoku Medical and Pharmaceutical University, 4-4-1 Komatsushima, Aoba-ku, Sendai 981-8558, Japan; rhenki@yahoo.com (H.R.); wdefny@yahoo.com (D.S.W.); deiske.sumilat@gmail.com (D.A.S.); booby_abdjul@yahoo.com (D.B.A.); wmaarisit@yahoo.com (W.M.); ky10nenb2@gmail.com (M.N.); 2 Graduate School of Pharmaceutical Sciences, Kitasato University, 5-9-1 Shirokane, Minato-ku, Tokyo 108-8641, Japan; ohtes@pharm.kitasato-u.ac.jp (S.O.); tomodah@pharm.kitasato-u.ac.jp (H.T.); 3 Faculty of Mathematic and Natural Sciences, Sam Ratulangi University, Kampus Bahu, Manado 95115, Indonesia; 4 Faculty of Fisheries and Marine Science, Sam Ratulangi University, Kampus Bahu, Manado 95115, Indonesia; 5 North Sulawesi Research and Development Agency, 17 Agustus Street, Manado 95117, Indonesia; 6 Faculty of Mathematics and Natural Sciences, Indonesia Christian University, Tomohon 95362, Indonesia; 7 Faculty of Nursing, University of Pembangunan Indonesia, Bahu, Manado 95115, Indonesia; magie_kapojos@yahoo.com; 8 Research Center for Genomic Medicine, Division of Biomedical Sciences, Saitama Medical University, 1397-1 Yamane, Hidaka, Saitama 350-1241, Japan; katagiri@saitama-med.ac.jp

**Keywords:** fibrodysplasia ossificans progressive (FOP), bone morphogenetic protein (BMP) signaling, alkaline phosphatase, screening, Indonesian marine sponge, *Dysidea* sp.

## Abstract

Fibrodysplasia ossificans progressiva (FOP) is a rare congenital disorder with heterotopic ossification (HO) in soft tissues. The abnormal activation of bone morphogenetic protein (BMP) signaling by a mutant activin receptor-like kinase-2 (ALK2) leads to the development of HO in FOP patients, and, thus, BMP signaling inhibitors are promising therapeutic applications for FOP. In the present study, we screened extracts of 188 Indonesian marine invertebrates for small molecular inhibitors of BMP-induced alkaline phosphatase (ALP) activity, a marker of osteoblastic differentiation in a C2C12 cell line stably expressing ALK2(R206H) (C2C12(R206H) cells), and identified five marine sponges with potent ALP inhibitory activities. The activity-guided purification of an EtOH extract of marine sponge *Dysidea* sp. (No. 256) resulted in the isolation of dysidenin (**1**), herbasterol (**2**), and stellettasterol (**3**) as active components. Compounds **1**–**3** inhibited ALP activity in C2C12(R206H) cells with IC_50_ values of 2.3, 4.3, and 4.2 µM, respectively, without any cytotoxicity, even at 18.4–21.4 µM. The direct effects of BMP signaling examined using the Id1WT4F-luciferase reporter assay showed that compounds **1**–**3** did not decrease the reporter activity, suggesting that they inhibit the downstream of the Smad transcriptional step in BMP signaling.

## 1. Introduction

Fibrodysplasia ossificans progressiva (FOP) is a rare genetic musculoskeletal disorder characterized by progressive and widespread postnatal heterotopic ossification (HO) in soft tissues [1,2,3]. The recurrent mutation R206H within ACVR1/ALK2, a subtype of bone morphogenetic protein (BMP) type I receptors, has been identified in FOP patients [4]. Previous studies reported that mutant ALK2 induced HO due to excess intracellular BMP signaling [5,6]. Accordingly, inhibitory substances toward BMP-induced osteoblastic differentiation (BMP signaling inhibitors) have potential in the treatment of FOP disease. Based on this concept, we established a cell-based assay system to evaluate alkaline phosphatase (ALP) activity, one of the markers of osteoblast differentiation, in stable ALK2(R206H)-expressing C2C12 cells (abbreviated as C2C12(R206H) cells) [7]. With this system, we identified a number of novel inhibitors from the culture broths of fungal and actinomycete strains [7,8,9,10,11].

To obtain additional structurally unique BMP signaling inhibitors, we have continued this screening program, with a focus on marine invertebrates (marine sponge and ascidian) as screening resources. The marine environment is a habitat for approximately 80% of all living organisms, and marine animals have developed individual metabolic abilities to survive under stressful conditions. Therefore, chemical studies on marine organisms have provided more than 25,000 new substances with diverse structural and biological features, most of which have not been obtained from terrestrial organisms [12,13].

In the present study, we screened 188 EtOH extracts of marine invertebrates collected in North Sulawesi in Indonesia for a new type of BMP signaling inhibitor. It is well known that Indonesia is rich in an enormous amount of bioresources.

**Figure 1:**
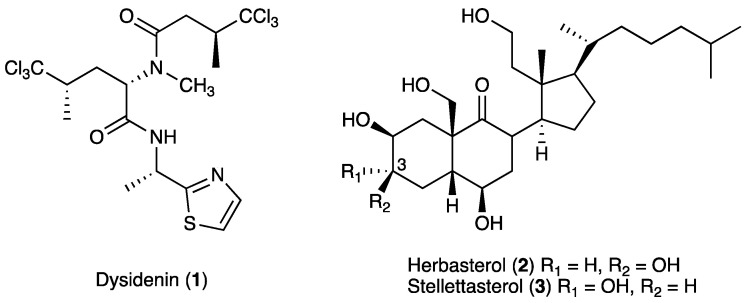
Structures of **1**–**3** from the Indonesian marine sponge *Dysidea* sp. (No. 256).

In the screening study, the EtOH extract of the marine sponge *Dysidea* sp. (No. 256) exhibited potent ALP inhibitory activity, and the bioassay-guided separation of the extract led to the isolation of one *N*- and *C*-substituted amino acid, dysidenin (**1**) [14,15,16], and two 9,11-secosteroids, herbasterol (**2**) [17] and stellettasterol (**3**) [18], as shown in Figure 1. We herein describe screening results as well as the isolation and biological activities of the compounds **1**–**3**.

## 2. Results and Discussion

We assessed the EtOH extracts of 188 Indonesian marine invertebrates, including marine sponges and ascidians, collected from Indonesian coral reefs in 2013 to define BMP signaling inhibitory activity using our established cell-based assay with C2C12(R206H) cells.

**Figure 2:**
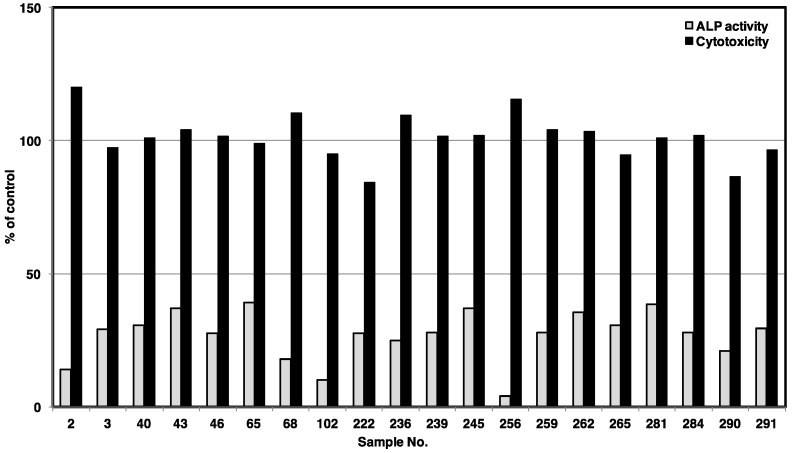
Alkaline phosphatase (ALP) inhibitory activities and cytotoxicities of selected marine invertebrate extracts in C2C12(R206H) cells. Cells were treated with marine invertebrate extracts at a concentration of 50 μg/mL. ALP activity and cell viability were measured on day 3.

The screening results showed that 20 marine sponge extracts inhibited ALP activity in C2C12(R206H) cells with >60% inhibition at 50 μg/mL, but no significant cytotoxicity in the MTT assay at the same concentration (Figure 2 and Table S1). Five out of the 20 extracts (Nos. 2, 68, 102, 256, and 290, shown in Figure S1) inhibited ALP activity by more than 80%. Among them, the marine sponge *Dysidea* sp. (No. 256), which exhibited the most potent activity (96% inhibition at 50 μg/mL), was investigated further to identify its active components.

The EtOH extract was purified using an ODS column followed by preparative HPLC (ODS) to give compounds **1** (1.7 mg), **2** (25 mg), and **3** (1.6 mg). Compounds **1**–**3** were identified as dysidenin [14], herbasterol [17], and stellettasterol [18], respectively, by comparing their spectroscopic data with those reported previously.

Compound **1**, originally isolated from the marine sponge *Dysidea harbacea* [14], was previously shown to exhibit inhibitory activities against the Na/I symporter [15] and lipoxygenase [16]. Compounds **2** and **3** were initially reported from the marine sponges *Dysidea* sp. and *Stelletta* sp., respectively. The fish toxicity of **2** [17] and antifungal activity of **3** [18] were demonstrated previously.

The BMP-induced ALP inhibitory activities and cytotoxicities of compounds **1**–**3** in C2C12(R206H) cells were measured using established methods [7,8,9,10,11,19] and their IC_50_ values are summarized in Table 1. Compound **1** exhibited ALP inhibitory activity in a dose-dependent manner with an IC_50_ value of 2.3 μM, and compounds **2** and **3**, epimers at C-3, inhibited ALP activity with similar potencies (IC_50_: 4.3 and 4.2 μM, respectively), as shown in Figure S2. Compounds **1**–**3** did not exhibit any cytotoxicity against C2C12(R206H) cells up to 18.4–21.4 μM in the MTT assay [18] (Table 1 and Figure S2).

The induction of ALP activity in C2C12 cells is the output of multiple intracellular events initiated by BMP [20]. BMP signaling is transduced via the transcriptional factors Smad1/5, which are phosphorylated and activated by BMP receptors [6]. Therefore, to examine the direct effects of compounds **1**–**3** on BMP signaling, a BMP-Smad specific Id1WT4F-luciferase reporter assay was performed [21].

The results obtained showed that compounds **1**–**3** did not affect luciferase activity, even at 18.4–21.4 μM, in this reporter assay (Table 1), suggesting that their molecular targets are downstream of the Smad transcriptional step. The modes of action of compounds **1**–**3** in osteoblastic differentiation currently remain unclear.

In conclusion, the screening of BMP signaling inhibitors from Indonesian marine invertebrates resulted in the discovery of three marine natural products, dysidenin (**1**), herbasterol (**2**), and stellettasterol (**3**), from the marine sponge *Dysidea* sp. (No. 256). This is the first study to report the BMP-induced ALP inhibitory activities of compounds **1**–**3**, and the results obtained provide useful information for understanding the relevant biological functions of BMP signaling and FOP.

## 3. Materials and Methods

### 3.1. Materials

The C2C12 myoblast cell line and the mutant C2C12(R206H) cell line were obtained from Prof. Takenobu Katagiri (Saitama Medical University, Saitama, Japan). Dulbecco’s modified Eagle’s medium was purchased from Nacalai Tesque (Kyoto, Japan). Fetal bovine serum was obtained from Capricorn (Ebsdorfergrund, Germany). Penicillin/streptomycin was obtained from Thermo Fisher Scientific (Waltham, MA, USA). p-Nitrophenyl phosphate was purchased from Sigma (St. Louis, MO, USA). Recombinant human BMP4 (rhBMP4) was obtained from R&D Systems (Mountain View, CA, USA).

The marine invertebrates used in the present study were collected by scuba diving at Manado and its surroundings, North Sulawesi, Indonesia in 2013. Voucher specimens were preserved in Sam Ratulangi University (Manado, Indonesia). Each sample was cut into small pieces and extracted with EtOH. After filtration, the solution was concentrated in vacuo to give a crude extract. The extracts obtained were dissolved in CH_3_OH at a concentration of 5 mg/mL and applied to the bioactive screening assay described below.

### 3.2. Isolation of Compounds ***1***–***3***

The marine sponge No. 256 was identified as *Dysidea* sp. The shape, appearance, and spicules and filaments detected under a microscope were very similar to those of the authentic specimen. A voucher specimen has been deposited at the Faculty of Mathematics and Natural Sciences, Sam Ratulangi University, as 13-12-14=2-256.

The sponge (67.6 g, wet weight) was cut into small pieces and extracted three times with EtOH (1 L) immediately after its collection. The EtOH extract (187 mg) was divided into six fractions (Frs. 1–6) with an ODS column (ODS CHROMATOREX (Fuji Silysia, Aichi, Japan), i.d. 15 × 60 mm) by stepwise elution with CH_3_CN in H_2_O. Compound **1** (1.7 mg) was isolated from Fr. 5 (23 mg, eluate with 80% CH_3_CN) by repeated HPLC (column, PEGASIL ODS SP100 (Senshu Scientific. Co., Ltd., Tokyo, Japan), i.d. 10 × 250 mm; solvent, 70% CH_3_CN in H_2_O containing 0.1% H_3_PO_4_; flow rate, 3.0 mL/min; detection, UV 210 nm). Fr. 3 (53 mg, first tube eluted with 60% CH_3_CN) was subjected to preparative HPLC (column; PEGASIL ODS SP100, i.d. 10 × 250 mm; mobile phase, 50% CH_3_CN in H_2_O containing 0.1% H_3_PO_4_; flow rate, 3.0 mL/min; detection, UV at 210 nm) to afford compound **2** (25 mg) and a subfraction (3.6 mg), which was further purified to give compound **3** (1.6 mg).

### 3.3. Cell Culture

The C2C12 myoblast cell line and the mutant C2C12(R206H) cell line [6] were cultured in Dulbecco’s modified Eagle’s medium supplemented with 15% fetal bovine serum, 100 units/mL penicillin, and 100 μg/mL streptomycin (hereafter referred to as medium A) at 37 °C in 5.0% CO_2_. C2C12(R206H) is more sensitive to BMP and exhibits stronger ALP activity than parental C2C12 cells. Both cell lines were subcultured once every three days.

### 3.4. Assay for ALP in BMP-Treated C2C12(R206H) Cells

ALP (Refseq: NP_001274101) activity, a typical marker of osteoblastic differentiation, was measured as previously described [7,8,9,10,11].

### 3.5. Cytotoxicity

The cytotoxicity of a compound to C2C12(R206H) cells was evaluated based on the 3-(4,5-dimethylthiazol-2-yl)-2,5-diphenyl tetrazolium (MTT) assay [19]. This assay was carried out according to our previous reports [7,8,9,10,11].

### 3.6. Reporter Gene Assay for Monitoring BMP Signaling

BMP signaling via Smad1/5 with the BMP-specific luciferase reporter (Id1WT4F-luc) was assayed by our established method [21].

## Figures and Tables

**Table 1:** Effects of **1**–**3** on osteoblastic differentiation, cytotoxicity, and BMP signaling.

Compound	IC_50_ (μM)		
	ALP ^a^	Cytotoxicity	BMP ^b^ Signaling
**1**	2.3	>18.4	>18.4
**2**	4.3	>21.4	>21.4
**3**	4.2	>21.4	>21.4

^a^ ALP: alkaline phosphatase; ^b^ BMP: bone morphogenetic protein.

## Supplementary Materials

The following are available online at https://www.mdpi.com/1660-3397/18/12/606/s1, Table S1: Collection dates and sites of Indonesian marine invertebrates selected for the screening of BMP signaling inhibitors. Figure S1: Pictures of Indonesian marine sponges with potent BMP-induced ALP inhibitory activity in C2C12(R206H) cells, Figure S2: Effects of **1**–**3** on ALP activities and cytotoxicities in C2C12(R206H) cells, Figure S3: ^1^H NMR (600 MHz, CD_3_OD) of dysidenin (**1**), Figure S4: ^1^H NMR (400 MHz, CD_3_OD) of herbasterol (**2**), Figure S5: ^1^H NMR (400 MHz, CD_3_OD) of stellettasterol (**3**).
