# Supplementary material for: Screening for Small Molecule Inhibitors of BMP-Induced Osteoblastic Differentiation from Indonesian Marine Invertebrates
Source: Mar Drugs. 2020 Nov 30;18(12):606. doi: 10.3390/md18120606 (PMC7761252; doi:10.3390/md18120606)
Supplement: Supplementary file 1 [file marinedrugs-18-00606-s001.pdf]

Marine Drugs
Supplementary Materials

Screening for Small Molecule Inhibitors of BMP Induced Osteoblastic Differentiation from Indonesian Marine Invertebrates

Hiroyuki Yamazaki, Satoshi Ohte, Henki Rotinsulu, Defny S. Wewengkang, Deiske A. Sumilat, Delfly B. Abdjul, Wilmar Maarisit, Magie M. Kapojos, Michio Namikoshi, Takenobu Katagiri, Hiroshi Tomoda, and Ryuji Uchida

Correspondence:
H. Yamazaki, Faculty of Pharmaceutical Sciences, Tohoku Medical and Pharmaceutical University, 4-4-1 Komatsushima, Aoba-ku, Sendai 981-8558, Japan.
E-mail: yamazaki@tohoku-pharm.ac.jp; Tel./Fax: +81-22-727-0218.

R. Uchida, Faculty of Pharmaceutical Sciences, Tohoku Medical and Pharmaceutical University, 4-4-1 Komatsushima, Aoba-ku, Sendai 981-8558, Japan.
E-mail: uchidar@tohoku-pharm.ac.jp; Tel./Fax: +81-22-727-0219.

Contents

^{1}H NMR data of **1–3**
References
Table S1 Collection dates and sites of Indonesian marine invertebrates selected by the screening of BMP signaling inhibitors.
Figure S1 Pictures of Indonesian marine sponges with potent BMP-induced ALP inhibitory activity in C2C12(R206H) cells.
Figure S2 Effects of **1**−**3** on ALP activities and cytotoxicities in C2C12(R206H) cells.
Figure S3 ^{1}H NMR (600 MHz, CD_3OD) of dysidenin (**1**).
Figure S4 ^{1}H NMR (400 MHz, CD_3OD) of herbasterol (**2**).
Figure S5 ^{1}H NMR (400 MHz, CD_3OD) of stellettasterol (**3**).

*^{1}H NMR data of **1–3***

Dysidenin (**1**)[1]: ^{1}H NMR (600 MHz, CD_3OD) δ 1.37 (3H, d), 1.41 (3H, d), 1.64 (3H, d), 2.02 (1H, m), 2.36 (1H, m), 2.58 (1H, t), 2.66 (1H, dd), 3.08 (3H, s), 3.21 (1H, d), 3.27 (1H, m), 5.35 (1H, q), 5.43 (1H, dd), 7.51 (1H, d), 7.72 (1H, d).

Herbasterol (**2**)[2]: ^{1}H NMR (400 MHz, CD_3OD) δ 0.78 (3H, s), 0.89 (6H, d), 0.95 (1H, m), 0.97 (1H, m), 1.01 (3H, d), 1.03 (1H, m), 1.16 (1H, m), 1.20 (1H, m), 1.37 (1H, m), 1.38 (1H, m), 1.40 (1H, m), 1.42 (1H, m), 1.43 (1H, m), 1.52 (1H, m), 1.54 (1H, m), 1.55 (1H, m), 1.57 (1H, m), 1.71 (2H, m), 1.82 (1H, m), 1.84 (1H, m), 2.09 (1H, m), 2.15 (1H, m), 2.53 (1H, ddd), 2.62 (1H, dd), 3.25 (1H, ddd), 3.34 (1H, ddd), 3.47 (1H, m), 3.53 (1H, d), 3.57 (2H, t), 3.80 (1H, brs), 4.70 (1H, d).

Stellettasterol (**3**)[3]: ^{1}H NMR (400 MHz, CD_3OD) δ 0.78 (3H, s), 0.89 (6H, d), 1.00 (3H, d), 1.03 (1H, m), 1.13 (2H, m), 1.14 (1H, m), 1.16 (2H, m), 1.19 (1H, m), 1.34 (1H, dd), 1.40 (1H, m), 1.43 (1H, m), 1.44 (1H, m), 1.50 (1H, m), 1.54 (1H, m), 1.55 (1H, m), 1.57 (1H, m), 1.68 (1H, m), 1.70 (1H, m), 1.79 (1H, m), 1.80 (1H, m), 2.08 (1H, m), 2.32 (1H, dd), 2.35 (1H, m), 2.52 (1H, ddd), 3.35 (1H, ddd), 3.52 (1H, d), 3.57 (2H, t), 3.69 (1H, m), 3.74 (1H, brs), 3.84 (1H, m), 4.75 (1H, d).

References

1. Kazlauskas, R.; Lidgard, R.O.; Wells, R.J.; Vetter, W. A novel hexachloro-metabolite from the sponge *Dysidea herbacea*. *Tetrahedron Lett.* **1977**, *36*, 3183–3186.
2. Capon, R.J.; Faulkner, D.J. Herbasterol, an ichthyotoxic 9, 11-secosterol from the sponge *Dysidea herbacea*. *J. Org. Chem.* **1985**, *50*, 4771–4773.
3. Li, H.; Matsunaga, S.; Fusetani, N. A new 9, 11-secosterol, stellettasterol from a marine sponge *Stelletta* sp. *Experientia* **1994**, *50*, 771–773.

Table S1. Collection dates and sites of Indonesian marine invertebrates selected by the screening of BMP signaling inhibitors.

No.	Date	Collection site
2, 3, 40, 43	December 9, 2013	Bualo near Manado Tua (around N1°36'48.168", E124°41'34.587")
46, 65, 68		Negeri near Manado Tua (around N1°36'53.575", E124°42'12.441")
102	December 10, 2013	Tanjung Pisok South (around N1°33'28.252", E124°47'43.328")
222, 236, 239	December 12, 2013	Boboca Statue near Malalayang (around N1°28'05.779", E124°49'22.555")
239		Kolonoom Beach near Malalayang (around N1°28'13.501", E124°49'29.042")
256, 259, 281, 284	December 13, 2013	Timur I near Bunaken (around N1°37'60.498", E124°48'21.160")
245, 262, 265, 290, 291		Pangalisang near Bunaken (around N1°36'07.372", E124°48'10.346")

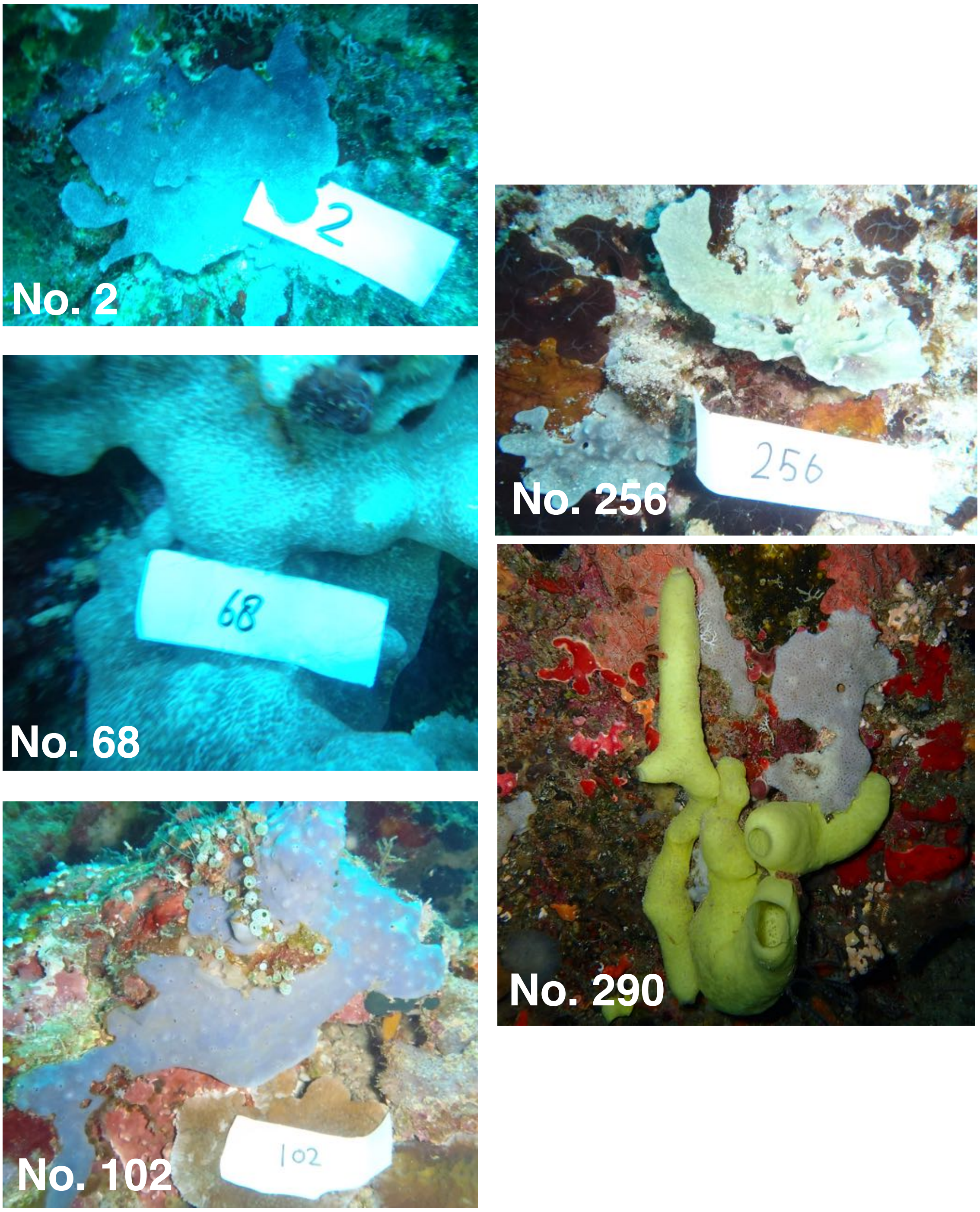

Figure S1 Pictures of Indonesian marine sponges with potent BMP-induced ALP inhibitory activity in C2C12(R206H) cells.

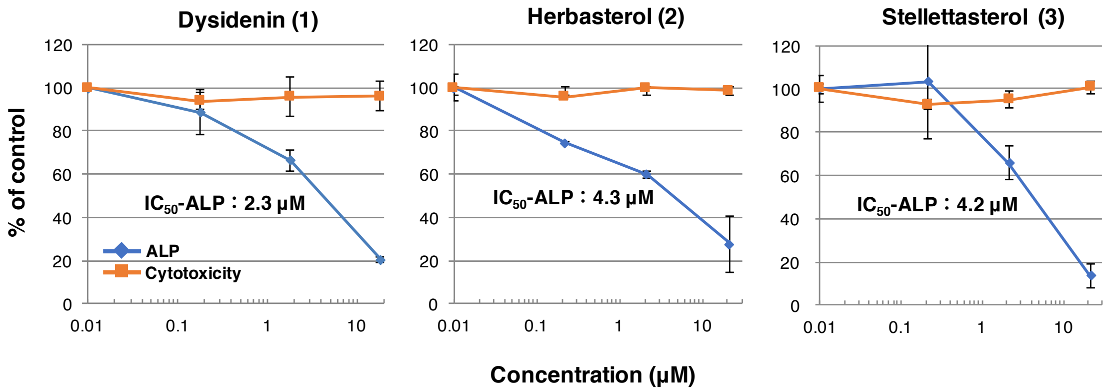

Figure S2 Effects of **1**−**3** on ALP activities and cytotoxicities in C2C12(R206H) cells. Cells were treated with each compound at the indicated concentrations. ALP activity and cell viability were measured on day 3.

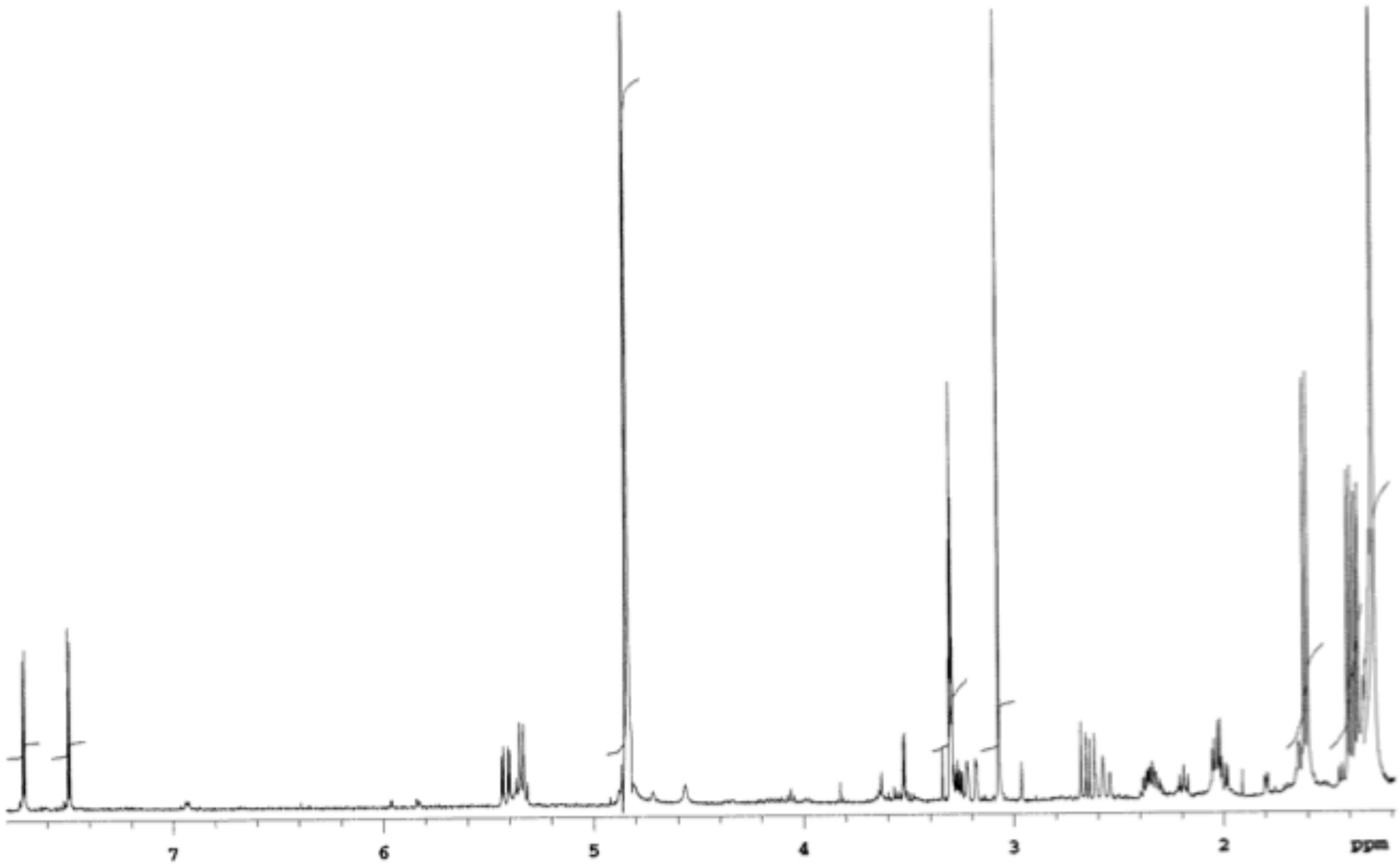

Figure S3 ^{1}H NMR (600 MHz, CD_3OD) of dysidenin (**1**).

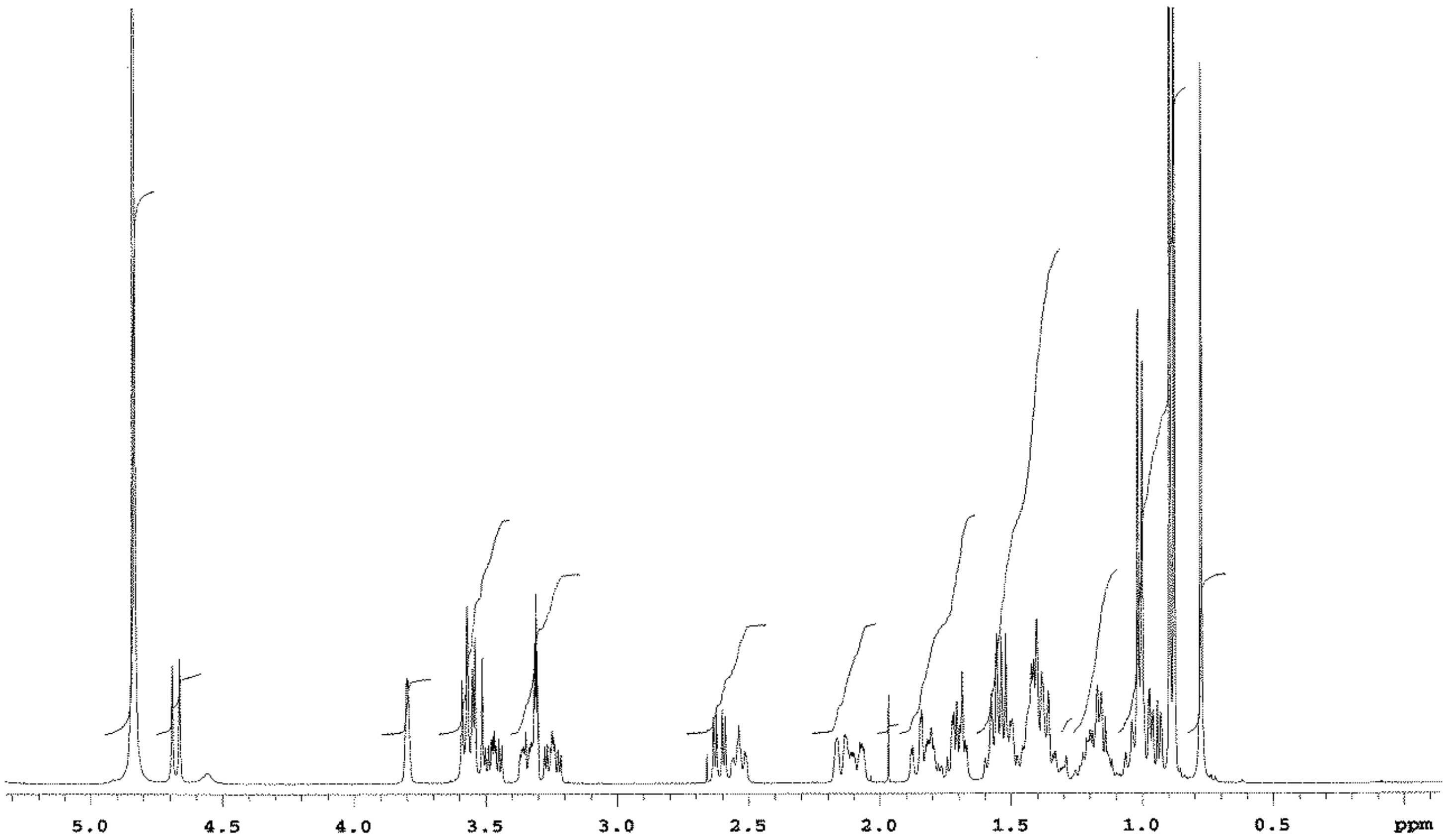

Figure S4 ^{1}H NMR (400 MHz, CD_3OD) of herbasterol (**2**).

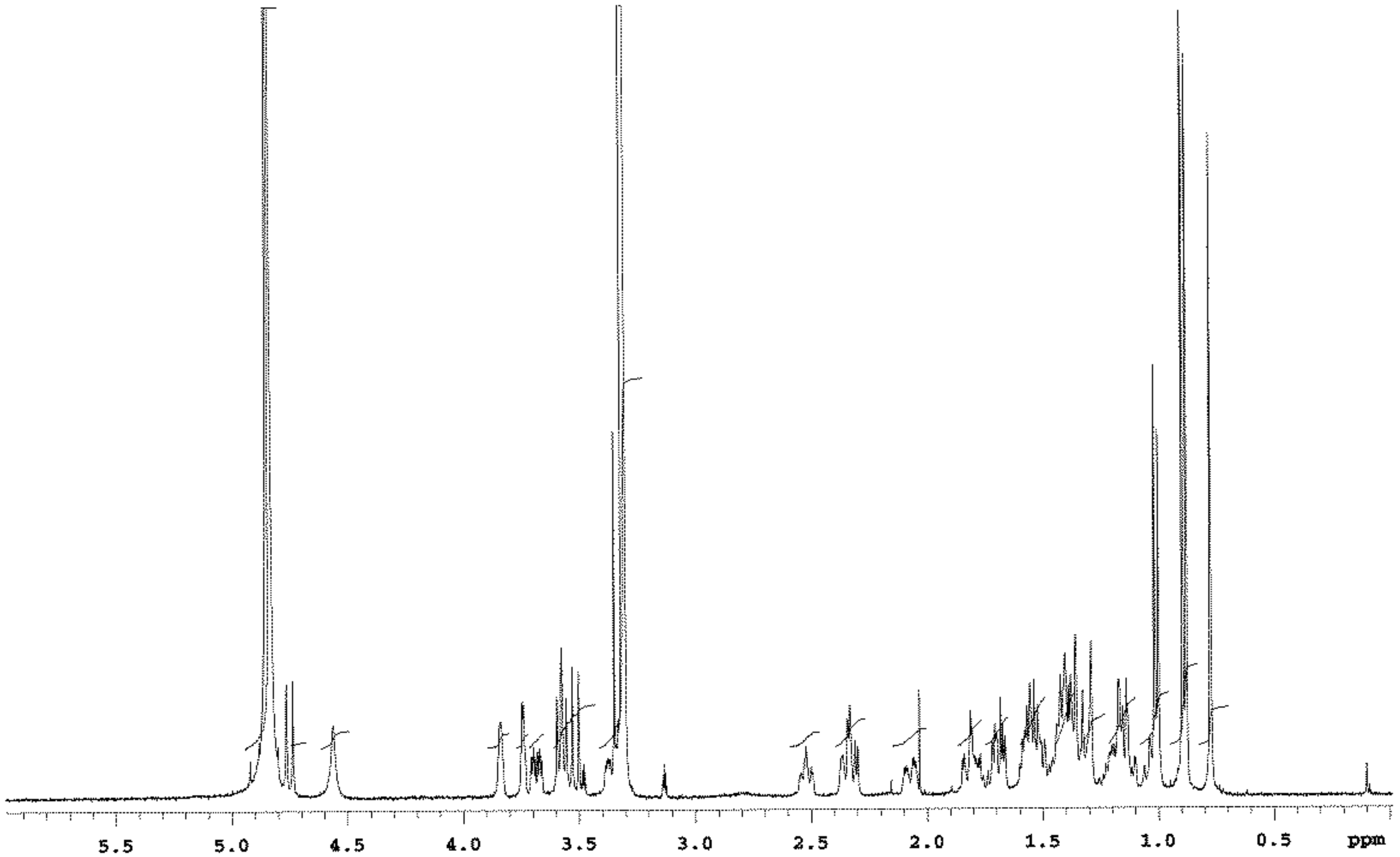

Figure S5 ^{1}H NMR (400 MHz, CD_3OD) of stellettasterol (**3**).
